# Just a Fad? Gamification in Health and Fitness Apps

**DOI:** 10.2196/games.3413

**Published:** 2014-08-04

**Authors:** Cameron Lister, Joshua H West, Ben Cannon, Tyler Sax, David Brodegard

**Affiliations:** ^1^LaughModel Health Communication Research GroupDepartment of Health ScienceBrigham Young UniversityProvo, UTUnited States

**Keywords:** gamification, mobile phone, behavioral health, health and fitness apps

## Abstract

**Background:**

Gamification has been a predominant focus of the health app industry in recent years. However, to our knowledge, there has yet to be a review of gamification elements in relation to health behavior constructs, or insight into the true proliferation of gamification in health apps.

**Objective:**

The objective of this study was to identify the extent to which gamification is used in health apps, and analyze gamification of health and fitness apps as a potential component of influence on a consumer’s health behavior.

**Methods:**

An analysis of health and fitness apps related to physical activity and diet was conducted among apps in the Apple App Store in the winter of 2014. This analysis reviewed a sample of 132 apps for the 10 effective game elements, the 6 core components of health gamification, and 13 core health behavior constructs. A regression analysis was conducted in order to measure the correlation between health behavior constructs, gamification components, and effective game elements.

**Results:**

This review of the most popular apps showed widespread use of gamification principles, but low adherence to any professional guidelines or industry standard. Regression analysis showed that game elements were associated with gamification (*P*<.001). Behavioral theory was associated with gamification (*P*<.05), but not game elements, and upon further analysis gamification was only associated with composite motivational behavior scores (*P*<.001), and not capacity or opportunity/trigger.

**Conclusions:**

This research, to our knowledge, represents the first comprehensive review of gamification use in health and fitness apps, and the potential to impact health behavior. The results show that use of gamification in health and fitness apps has become immensely popular, as evidenced by the number of apps found in the Apple App Store containing at least some components of gamification. This shows a lack of integrating important elements of behavioral theory from the app industry, which can potentially impact the efficacy of gamification apps to change behavior. Apps represent a very promising, burgeoning market and landscape in which to disseminate health behavior change interventions. Initial results show an abundant use of gamification in health and fitness apps, which necessitates the in-depth study and evaluation of the potential of gamification to change health behaviors.

## Introduction

### Mobile Phone Technology and Health Behavior Change

Mobile phone technology has recently become an area of focus for disseminating health behavior change interventions [1-3]. This technology has the capacity to provide for easy collection of personal health-related data and providing timely behavioral cues [4,5]. Additionally, research has focused on the benefits of mobile and Internet technologies for reaching diverse populations to reduce health disparities [6,7], and rural communities for health interventions [8]. Some of the most recognizable research has focused on text messaging interventions, or short message service (SMS) [9]. This technology has been used to study several health topics like physical activity [10], diabetes self-management [11], and smoking cessation [12].

Since 2007 when Apple introduced the iPhone, followed by Google’s Android, mobile phone sales have outpaced those for conventional cell phones; 56% of Americans now own a mobile phone [13]. Third party apps are software programs that serve to expand the utility of mobile devices. Within just 6 years, Apple celebrated its 50 billionth app download, with Google only trailing slightly behind with 48 billion as of May 2013 [14]. This new market of software apps for Apple alone has resulted in over US $9 billion being paid to developers [14]. Health apps have also become a part of this market, with over 31,000 health and medical apps available for download [2]. With mobile phone ownership and the number and complexity of health apps likely to increase, the potential for technology-based health interventions to impact populations is expanding in ways previously not possible.

### Gamification

The term “gamification” originally coined in 2008, and later broadly used by technology and health professionals through the first half of 2010, encompasses a broad spectrum of technology and game-like elements into the commercial world [15]. For functional purposes, the definition formalized by Deterding et al, will be used throughout the remainder of this paper. This definition states “gamification is the use of game design elements in nongame contexts” [15]. Companies have widely accepted and adopted gamification as a means to increase initiation and retention of desired behaviors [16], additionally it has been estimated that 60% of health initiatives in workplaces now include gamification elements [17,18]. Furthermore, gamification is on track to becoming a 2.8 billion dollar industry by the year 2016, with little to no evidence in the scientific literature as to its efficacy in improving desired outcomes in regards to health and health behaviors [19].

Gamification in mobile app technology has emerged as a popular strategy, both in commercial culture and the field of academia as a means of influencing behaviors [15,16,20,21]. Gamification is the use of game-like rewards and incentives, paired with desired behaviors, to increase motivations and sustain habits of individuals over time [15,20]. The use of this tactic in health and fitness mobile apps has increased despite little to no in-depth inquiry into its effectiveness and appropriate functionality [17,18]. The purposes of this study were to review health and fitness apps for elements of gamification, to determine the relationship between health app use of gamification and core elements of effective games, and to determine the extent to which apps with gamification elements contain critical health behavior constructs.

## Methods

### Study Design and Background

This study design involved an analysis of gamification and health behavior constructs of Apple iPhone health and fitness apps beginning in the winter of 2014. Each app was measured for elements of gamification and health behavior, as explained below in the subsection Measures. Research assistants were recruited from undergraduate and graduate health science students at a midwestern university. The research assistants were trained in the rubrics for gamification and health behavior constructs as seen in Tables 2 and 3.

### Sample Identification

The sample was collected from the Apple App Store in the winter of 2014. This sample contained apps that are under the health and fitness section of the App Store and related to the particular health behaviors of diet and physical activity. There were 40 key search terms that were established prior to the sample collection using key phrases for both physical activity and diet apps that may contain gamification; keywords included, “running”, “walking”, “health games”, “gamification”, “diet”, “calorie counting”, and others related to these behaviors (see Table 1). The study’s authors selected the search terms and have formal training in public health and health behavior. Search terms were entered into the Apple App Store on the most recent version of the iPad, due to the fact that iPads allow the user to filter search results. Search results were narrowed by: (1) iPhone Only, (2) Free Apps, (3) Health and Fitness, and (4) Popularity. According to most recent estimates, 90% of apps available in the Apple App Store are free, representing a growing trend toward a *freemium* platform, or in-app purchases for upgrades; for this reason only free apps were included in the sample generation, as they are most representative of the majority of available apps [22]. Additionally, previous studies reviewing the content of apps also ordered search results by popularity to ensure that the apps that were reviewed were highly used [23].

**Table 1:** Apple App Store search terms.

Physical activity search terms	Diet search terms	Gamification search terms
1) running	1) burn calories	1) games for health
2) jogging	2) diet	2) healthy games
3) walking	3) calories	3) health games
4) cross training	4) calorie counter	4) games
5) exercise	5) healthy diet	5) gamification
6) workout	6) diet tracker	6) gamified health
7) work out	7) healthy food	7) health challenge
8) aerobics	8) healthy eating	
9) trainer	9) carbs	
10) weight lifting	10) carb tracker	
11) cycling	11) carb counter	
12) fitness	12) lose weight	
13) health coach	13) BMI^a^	
14) cardio	14) healthy weight	
15) weight training		
16) fitness class		
17) health class		
18) aerobics class		
19) sports		

^a^BMI=body mass index

### Coding Procedure

The detailed written descriptions for the first 20 apps that appeared in the search results under each topic were read and coded into an initial sampling rubric using Qualtrics online survey software. This was done in order to ensure that the apps that were downloaded and reviewed for the final analysis contained at least one component of gamification. The initial rubric contained basic descriptive information (name of the app, number of reviews, price, year of development, and whether the app pertained to health or a health behavior) and the six core components of gamification outlined by the public health literature, and explained below in the subsection Measures. Since the Apple App Store does not sort search results by page numbers, a set number was established for each search term; additionally, other sampling criteria in search engines has established that looking through a set number of primary results (ie, 1 to 2 pages) is enough to establish the quality of sampling, as users are unlikely to go beyond the first page of results [24,25].

A total of 800 apps were returned for preliminary coding. Some search terms yielded no relevant results as to the purposes of the study, so the results were not coded for that entire search term; for example, if none of the app descriptions contained any components of gamification, or if no search results were brought up. After eliminating duplicates, a total of 261 app descriptions were coded for inclusion in the final sample. In order to ensure sample quality, the sample was refined by using apps that were initially coded as having at least one of the six core components of gamification. After applying this criteria for inclusion, 129 apps were excluded from the sample for having no components of gamification, leaving a total of N*=*132 apps for a final analysis of apps using gamification.

### Interrater Reliability

A hard-copy version of the survey with explanations of each individual game component and behavioral construct was supplied to each of the coders in order to provide a common reference for defining each individual term (see Tables 2 and 3). Each assistant independently coded an identical 10 percent of the sample in order to establish interrater reliability. A kappa coefficient was used to measure reliability between the three coders, a well established method that has been used in similar studies [23,26]. After coding an identical 10.6% of the initial sample (14/132), a kappa coefficient of 0.66 was measured between the coders. This is rated as a substantial level of agreement between coders, and is an acceptability measure of reliability for this study [27].

**Table 2:** Construct definitions and app functionality.

Behavioral constructs					Construct definition		Example function on app				Behavioral model/theory
**Capacity**											
	**Psychological**										
			General information		Information designed to increase basic knowledge about the behavior.			Information about Centers for Disease Control and Prevention guidelines for exercising. (Ex. 2 hours and 30 minutes of moderate aerobics per week)			HBM^a^, TTM^b^, TPB^c^, SCT^d^
			Self-monitoring		Behavioral tracking to increase the ability of the individual to make informed decisions.			Info-graphs, summaries of charts, and trends over time.			TTM^b^, SCT^d^, Fogg^e^
			Stress management		Improving the emotional and mental ability to cope with change and make strategic changes in an individual’s life.			Information on how to cope with stress of changing your diet.			SCT^d^
	**Physical**										
			Skills training		Providing training to increase the physical ability of an individual to perform a behavior.			Instructional video/tutorial on a new lifting technique or a connection to a trainer.			TTM^b^, SCT^d^
			Simplicity or enabling factors		Things that serve to make the behavior easier to accomplish by eliminating barriers or making a task simple. (time, money, physical effort, etc)			Time management tools or money saving tools to help engage in the behavior more frequently.			SCT^d^, PPM^f^, Fogg^e^
**Motivation**											
	**Automatic**										
			Incentivization (rewards)		Based in operant conditioning, pairing the behavior with rewards or incentives to train an individual to value the behavior.			Gaining points that can be cashed in for a monetary prize, or creating a self-reward.			TTM^b^, SCT^d^
			Social support (positive reinforcement)		Pairing the behavior with support from new or old social spheres that provide validation and positive reinforcement with new behavioral changes.			Sharing information on social media for comments, discussion boards, and adding friends on an app interface.			TTM^b^, SCT^d^, PPM^f^, Fogg^e^
	**Reflective**										
			Goal-setting		Creating small attainable goals to help individuals begin new behaviors and keep commitments.			Setting goals to run 3 times a week for 30 minutes.			SCT^d^, Fogg^e^
			Cognitive strategies		Perceived benefits, barriers, risks, severity, and social norms. Information about performing the behavior, and questions and discussions to help individuals evaluate beliefs.			A discussion board that prompts key questions related to the behavior.			HBM^a^, TTM^b^, TPB^c^, SCT^d^, PPM^f^
			Self-efficacy		Creating a self-mastery experience or using modeling/ vicarious learning to help improve an individual’s confidence in doing the behavior.			Breaking the behavior into small attainable steps, notification of peers doing the correct behavior.			TTM^b^, TPB^c^, SCT^d^, Fogg^e^
**Opportunity/trigger**											
	**Social**										
			Peer pressure		Using peers to enforce new rules about behaviors or changing social settings/context to eliminate negative influences on behavior.			Competitions or functions to encourage friends to achieve their goals. Shared accountability.			TTM^b^, SCT^d^, Fogg^e^
	**Physical**										
			Cues to action		Cues from the physical environment that help eliminate the need to depend on memory, and prompt needed action. (ecological momentary assessment).			Reminders through push notifications to go running.			HBM^a^, Fogg^e^, PPM^f^
			Stimulus control		Restructuring your environment to eliminate bad triggers and increase positive influences.			Setting constraints on unhealthy food in the house, limiting screen-time.			TTM^b^, SCT^d^, Fogg^e^

^a^HBM=health belief model

^b^TTM=transtheoretical model

^c^TPB=theory of planned behavior

^d^SCT=social cognitive theory

^e^Fogg=BJ Fogg model of behavior

^f^PPM=precede-proceed model

### Measures

Each app was coded for general information, the 10 effective elements of games, 6 core components of gamification for health, and 13 core health behavior constructs. Given the above definition of gamification being “the use of game design elements in nongame contexts”, the rubric for components of gamification was similar to the rubric for game elements. This is due to gamification being adopted from the field of videogames; however, as shown below in Table 3, the rubric for game elements is more extensive and includes the “game context”. For the purposes of this paper, the 10 effective elements of games were taken from the current literature in the field of gaming, or rather the model of set standards for games set by professionals in the video gaming industry [28]. The six core components of gamification were taken from the behavioral and public health literature as defined by health professionals. General information included number of app reviews, use of external resources as citations (content validity), method of data collection (passive or active), target health behavior, app integration with other technologies, and perceived purpose of gamification. It should be noted that the number of app reviews was used as a crude measure of the popularity of the app, as the apps with the most reviews generally were reviewed more favorably, and likely received more downloads than other apps. Additionally, the search results were ordered according to the popularity in the app store (as detailed above), with the top results always having the largest number of reviews.

Coding of the 10 effective game elements was taken from the work of Reeves and Read, which outlined the ten effective elements of games, and quoted in the work of Deterding et al [15,28]. These elements included the following: (1) self-representation with avatars; (2) three dimensional environments; (3) narrative context (or story); (4) feedback; (5) reputations, ranks, and levels; (6) marketplaces and economies; (7) competition under rules that are explicit and enforced; (8) teams; (9) parallel communication systems that can be easily configured; and (10) time pressure (see Table 3). These components were coded as either 1=present and used in the app, or 0=not present.

The six gamification components were determined by reviewing the current body of literature and finding common themes and components of gamification used or discussed in the literature for impacting health behavior [15,17,18]. The same coding procedure as outlined above was used to code the gamification components, which included: (1) leaderboards, (2) levels, (3) digital rewards (points, badges), (4) real-world prizes, (5) competitions, and (6) social or peer pressure (see Table 3).

The 13 health behavior constructs were identified from the combined work of Doshi et al, Cowan et al, and Michie et al [23,29,30]. Doshi et al established a rubric for evaluating physical activity websites for health behavior constructs that included 20 constructs from the most common behavior models in use in public health practice, health belief model (HBM), transtheoretical model, theory of planned behavior, and social cognitive theory (SCT) [29]. Cowan et al, built on this work by applying the same rubric to physical activity apps [23]. However, not all of the constructs are applicable to mobile apps, and many of the constructs have similar overlapping definitions and components. Due to these limitations, a new rubric was compiled and consolidated for clarity. Additionally, work by Michie et al outlined the “Behavior Change Wheel” for conceptualizing behavior change interventions. This framework establishes health behavior as having 3 main components: (1) capability (psychological and physical), (2) motivation (automatic and reflective), and (3) opportunity/trigger (social and physical) (COM-B) [30]. Similar conceptualizations and iterations of this same model exist in precede-proceed model and the BJ Fogg model of behavior [31,32]. The 20 constructs from Doshi et al and Cowan et al were categorized based on these components from the COM-B system and consolidated.

The final rubric contained the following indicators for measurement for a total of 13 constructs (see Table 2); capability (Psychological, general information, self-monitoring, stress management; Physical, skills training, simplicity or enabling factors), motivation (Automatic, incentivization, social support; Reflective, goal-setting, cognitive strategies from HBM, self-efficacy), and opportunity/trigger (Social, peer pressure; Physical, cues to action, stimulus control). Constructs were included if they had clear distinguishable definitions, and if they had direct application to functions of an app on a mobile phone (Table 2). In order to eliminate subjective bias, the behavioral constructs were coded and scored the same as the game and gamification rubrics (Yes=1, No=0).

### Data Analysis

Descriptive statistics were used to report on the integration of gamification components into health and fitness apps. After coding each individual app for effective game elements, gamification components, and health behavior constructs, a final score was totaled for each category. Linear regression analysis was used to test the remaining two hypotheses. The first regression assessed the association between game elements and total gamification components; integrating the total opportunity/trigger score, app reviews, target health behavior, and app integration with other technologies into the model. The second regression assessed the total gamification components with the three individual subbehavioral scores, capacity, motivation, and opportunity/trigger; using the purpose of gamification and app integration with other technologies integrated into the model. The final regression compared the total behavioral construct score to total game elements and total gamification components; integrating the number of app reviews, target health behavior, content validity, method of data collection, and app integration with other technologies into the model.

## Results

### Descriptive Statistics

Of the originally coded sample of 261 app descriptions, 52.5% (137/261) contained at least one element of gamification, with around 23.8% (62/261) containing at least half (3 or more) of the 6 most commonly used elements of gamification in health. Social or peer pressure was the most common element of gamification used, with just over 45.2% (118/261) of apps containing this component, followed by digital rewards, competitions, leaderboards, level of achievement or rank, and real world prizes (24.1%, 63/261; 18.4%, 48/261; 14.2%, 37/261; 13.4%, 35/261; and 10.0%, 26/261 respectively). Of the apps coded, a total of 88.5% (231/261) of the sample pertained to a health behavior, while the remaining 11.5% (30/261) were either primarily educational, or not focused on health behavior change.

Of the 132 apps that were downloaded and coded in the final comprehensive analysis, 68.2% (90/132) were exclusively for physical activity, 9.1% (12/132) were for dietary tracking and behavior, 19.7% (26/132) were comprehensive or both physical activity and diet, with 3.0% (4/132) targeting other health behaviors. There were 91.7% (121/132) of the apps that contained no citations or links to sources to verify the information provided in the app, and 29.5% (39/132) of the apps were found to integrate with other technologies or media. Around 97.7% (129/132) of the apps tracked some sort of data from the user, with 27.9% (36/129) tracking in passively (not requiring manual input of data), 55.0% (71/129) having active tracking (requiring the user to manually input data), and 17.1% (22/129) using both methods. Finally, coders rated the perceived purpose of gamification use in the apps. There were 14.4% (19/132) of the perceived apps’ purpose that were coded as “to get people to interact with the app more”, 32.6% (43/132) were coded as “to get people to do more completions of the desired behavior”, 43.2% (57/132) were coded as both, and 9.8% (13/132) were coded as neither or “purpose unclear”.

The 132 apps included in the sample had a mean behavioral score of 4.99 out of 13 possible (38.4%; Cronbach alpha=.65), a mean game score of 3.80 out of 13 possible (29.25%; Cronbach alpha=.72), and a mean gamification score of 2.28 out of 6 possible (38.13%; Cronbach alpha=.64). The behavioral score was further broken down into three components (as explained above): (1) capability (mean 2.11 of 5; 42.12%), (2) motivation (mean 2.30 of 5; 46.06%), and (3) opportunity/ trigger (mean 0.58 of 3; 19.4%). Table 3 shows detailed descriptive statistics for each of the components of the 3 measuring rubrics designed for this study.

**Table 3:** Descriptive statistics for evaluation rubrics (N=132).

Measuring rubrics			N=132 (number of apps)	%	Mean	Median	SD
**Behavioral**			-	-	4.99	5.0	2.44
	**Capacity**		-	-	2.11	2.0	1.1
		General information	51	38.64	-	-	-
		Self-monitoring	129	97.73	-	-	-
		Stress management	19	14.39	-	-	-
		Skills training	42	31.82	-	-	-
		Simplicity or enabling factors	37	28.03	-	-	-
	**Motivation**		-	-	2.30	2.0	1.58
		Incentivization	32	24.24	-	-	-
		Social support (positive reinforcement)	54	40.91	-	-	-
		Goal-setting	75	56.82	-	-	-
		Cognitive strategies	68	51.52	-	-	-
		Self-efficacy	75	56.82	-	-	-
	**Opportunity/trigger**		-	-	0.58	0.0	0.68
		Peer pressure	48	36.36	-	-	-
		Cues to action	25	18.94	-	-	-
		Stimulus control	4	3.03	-	-	-
**Game elements**			-	-	3.80	3.5	2.68
	Self-representation with avatars		68	51.52	-	-	-
	3-D environments		8	6.06	-	-	-
	Narrative context		8	6.06	-	-	-
	Feedback from game (before or during)		45	34.09	-	-	-
	Feedback, reinforcement (after)		76	57.58	-	-	-
	Leaderboards		43	32.58	-	-	-
	Ranks of achievements		39	29.55	-	-	-
	Different levels of play		28	21.21	-	-	-
	Marketplaces and economies		19	14.39	-	-	-
	Competition under rules explicit and enforced		42	31.82	-	-	-
	Teams (multi-player modes)		19	14.39	-	-	-
	Parallel communication systems		64	48.48	-	-	-
	Time pressure		43	32.58	-	-	-
**Gamification**			-	-	2.29	2.0	1.66
	Leaderboards		43	32.58	-	-	-
	Levels of achievement or rank		34	25.76	-	-	-
	Digital rewards		73	55.30	-	-	-
	Real world prizes		24	18.18	-	-	-
	Competitions/challenges		51	38.64	-	-	-
	Social or peer pressure		78	59.09	-	-	-

### Linear Regression Analysis

Results from the regression analysis (Tables 4-6) showed that greater inclusion of game elements was significantly associated with gamification components (*P*<.001), and was associated with the total opportunity/trigger score (*P*<.05). Second, inclusion of gamification components in app design was found to be significantly associated with total motivation score (*P*<.001), while showing no correlation to total capacity score or total opportunity score. Finally, the regression showed that inclusion of behavioral theory in apps was associated with inclusion of gamification components (*P*<.05), but was not associated with inclusion of game elements. Additionally, behavioral theory was positively associated with number of app reviews (*P*<.05), the targeted health behavior (*P*<.05), and was associated with method of data collection (*P*<.05).

**Table 4:** Regression analysis, for total game elements (N=132).^a^

Variable	DF	Type III sum of squares	Mean square	*F* value	*P*> *F*
Total behavioral score	1	0.2226962	0.2226962	0.09	.7661
Total gamification score	1	316.6222861	316.6222861	126.33	<.001^b^
Total opportunity score	1	13.1144394	13.1144394	5.23	.0239^c^
Number of app reviews	1	15.5375399	15.5375399	6.20	.0141^c^
Target health behavior	3	3.2131914	1.0710638	0.43	.7337
App integration with other technologies	1	6.9311679	6.9311679	2.77	.0989

^a^Number of observations used=132. *r* ^*2*^=.6717

^b^ *P* value<.001

^c^ *P* value<.05

**Table 5:** Regression analysis, for total gamification components (N=132).^a^

Variable	DF	Type III sum of squares	Mean square	*F* value	*P*> *F*
Total motivation score	1	24.17913828	24.17913828	28.94	<.001^b^
Total capacity score	1	0.02400399	0.02400399	0.03	.8657
Total opportunity score	1	1.73260282	1.73260282	2.07	.1524
Total game score	1	91.61451097	91.61451097	109.66	<.001^b^
Purpose of gamification	3	6.58606083	2.19535361	2.63	.0533
App integration with other technologies	1	0.00144093	0.00144093	0.00	.9669

^a^Number of observations used *=*132. *r* ^*2*^=.7169

^b^ *P* value<.001

**Table 6:** Regression analysis, for total behavioral constructs (N=132).^a^

Variable	DF	Type III sum of squares	Mean square	*F* value	*P* > *F*
Total game score	1	9.46638187	9.46638187	3.07	.0825
Total gamification score	1	26.78349544	26.78349544	8.68	.0039^b^
Number of app reviews	1	35.73079259	35.73079259	11.57	.0009^b^
Target health behavior	3	49.69643792	16.56547931	5.37	.0017^b^
Content validity (citations)	1	5.73648830	5.73648830	1.86	.1754
Method of data collection on app	2	27.95652683	13.97826341	4.53	.0127^b^
App integration with other technologies	1	0.08936760	0.08936760	0.03	.8652

^a^Number of observations used *=*129. *r* ^*2*^ *=*.4946

^b^ *P* value<.05

## Discussion

### Gamification Use

This research represents, to our knowledge, the first comprehensive review of gamification use in health and fitness apps. The results show that use of gamification in health and fitness apps has become common, as evidenced by the number of apps found in the app store containing at least some components of gamification. The use of game elements was correlated with the use of gamification, which was expected as gamification borrows its constructs from the gaming space. Gamification scores correlated with the use of health behavior theory, although further analysis showed that only motivation accounted for the association. This was expected as gamification in the industry is generally used to increase the motivation of its users; however, this potentially highlights a missed opportunity with gamification apps focusing primarily on motivational components of behavior without adequately addressing capability or behavioral triggers.

Despite the inclusion of at least some components of gamification, the mean scores of integration of gamification components were still below 50 percent. This was also true for the inclusion of game elements and the use of health behavior theory constructs, thus showing a lack of following any clear industry standard of effective gaming, gamification, or behavioral theory in health and fitness apps. Moving toward an industry standard may be challenging, however, as it is difficult to measure the true impact of gamification without conducting experiments related to the impact of design features of apps. In large part there has been little effort for ensuring quality in commercial health and fitness apps or establishing effective and meaningful criteria for using gamification. In fact, the current success of health games is measured in revenue generation, not behavioral metrics [20,33,34]. Much research on gamification and health to date has focused on exergames, or games that require the individual to be physically active while playing the game [35]. Gaming consoles like the Nintendo Wii or Xbox Kinect have used interactive sensors to allow for more integrated types of game play that were not before possible [17,34]. Work by Adams et al has focused on developing evaluation frameworks for effective components and physical exertion in exergames [35]. The results of this study show the need for further examination of games in health through large sample studies in controlled settings in order to measure the true benefits of gamification for health.

As hypothesized, use of gamification was correlated with effective game elements established by the videogame industry. Additionally, game elements were correlated to app popularity, as represented by the number of app reviews. Considering the Apple App Store does not report data on popularity through downloads or consistent use of particular apps, reviews were the only quantifiable measure of popularity universally available for inclusion in analysis. In contrast, gamification was not correlated to app popularity when placed in the regression analysis (not reported in tables). This has significant implications for developers and health practitioners when designing games for health. If games are in fact more popular to the public, then the focus of developers should be to create in-depth, narrative gaming experiences and not apps that merely use convenient elements of games or gamification.

Much controversy has developed over the broadly used term, gamification. The widespread adoption of gamification in health apps has been criticized from the field of game developers, as it only adopts selective or “convenient” components of functional games into nongame settings [15,18,36]. This adaptation removes the fact that the traditional games are already naturally reinforcing and motivating, while complex behaviors such as diet and physical activity may not be [18,33,34]. Ferrara referred to the work of Gartner when describing this rapid expansion and widespread adoption of gamification as being a part of a naturally reoccurring “hype” cycle that occurs with new technologies, and later dies down after increasing amounts of failure and frustration from the field [18,37]. Additionally, the push towards gamification assumes that the use of rewards, levels, leaderboards, and external incentives are enough to sustain (health) behavior responses without using other components of games like problem solving, storytelling, and fantasy [15,18,36]. This mirrors the findings of this study, with results showing very little use of 3-D environments and narrative context.

Finally, the results of this analysis showed that the use of health behavior theory was correlated with the use of gamification and not the use of game elements. Further analyses showed that gamification correlated with the composite motivation score and not to capacity or opportunity/trigger. Perhaps apps that use gamification are trying to influence the motivation of users to engage in a desired behavior, while potentially ignoring the ability of an individual to perform the behavior and triggers to engage in a behavior. However, targeting ability of individuals to engage in a behavior is central to achieving long-term behavioral change [30]. A singular focus on motivation may temporarily support health behaviors, but without increased ability, motivation and self-efficacy may not be sustained [32,38].

While the new field of gamification is promising, it has overlooked other major components of health behavior theory in the development and implementation of gamified health. First, the concept of using incentives to increase adoption and retention of behaviors finds its origins in operant conditioning/ reinforcement, or repetitive pairing of completion of the behavior with an external reward [35,38]. However, no attempt has been made to assess the use of gamification in mobile phone apps as it relates to operant conditioning. This review of gamification in health apps reveals that many apps rely on the digital rewards such as badges or points as being valued by the user, when in actuality, this may not be true. This perspective is not supported in traditional behavior change settings, with some professionals criticizing this use of gamification as being little more than a customer loyalty program not geared for comprehensive behavioral change [18]. Second, no assessment has been made as to whether or not external incentives in mobile phone apps adequately adhere to best practice in health behavior theory application. Indeed, many apps require too much effort in engagement for too little perceived worth or benefit, an imbalance that results in unreliable health behaviors [35,39,40].

### Limitations

The findings of this study should be interpreted in the context of some key limitations. First, primarily free apps were reviewed in the sample of this study, which may have excluded relevant available paid apps that may use gamification. However, a growing trend over recent years has been toward making apps free to download, with 90% of apps currently being free [22]. As such, this sample should be considered sufficient representation of apps available considering this industry trend.

Second, apps were originally coded into the sample by reviewing the descriptions in the Apple App Store. Consequently, some of the apps initially reviewed (around 16.7%, 22/132) did not actually include elements of gamification as previously coded. However, this potential error came after complex review of apps after download, and was the result of inaccurate descriptions, not the researchers of this study. Further emphasis in the app industry should move toward quality control of descriptions and app content in order to provide better quality content to users. Finally, this study did not evaluate the role of design features of apps in effecting their behavioral efficacy. As such, the results should be interpreted with caution, and used to lay a foundational framework for developing future studies that can incorporate elements of app design in their evaluation; for instance, randomized controlled trials.

### Conclusions

Apps represent a very promising, burgeoning market and landscape in which to disseminate health behavior change interventions. Initial results show an abundant use of gamification in health and fitness apps, which necessitates the in-depth study and evaluation of the potential of gamification to change health behaviors. Developers and health practitioners trying to influence behavior change and health outcomes should consider comprehensive integration of behavioral theory, independent of whether or not games or gamification is used. However, gamification may be an effective means of targeting motivational components, and games may be effective at triggering individuals and increasing popularity of apps. As it stands, the current industry use of gamification, game elements, and behavioral theory are subpar, illustrating a proliferation of apps available for download following no set industry standard that is currently available. This paper has the potential to not only impact the burgeoning industry of gamification in health and fitness apps, but to provide a framework for effective practice of integrating games and behavioral theory into mobile interventions to better impact the health of populations.

## Abbreviations

COM-B: capability motivation opportunity/trigger
HBM: health belief model
SCT: social cognitive theory
SMS: short message service
